# Hydrogel delivering self-assembled herbal nanoparticles accelerates diabetic wound healing through mitochondrial regulation

**DOI:** 10.1016/j.mtbio.2025.102417

**Published:** 2025-10-17

**Authors:** Jiahe Guo, Ben Hu, Yi Wei, Guopan Cheng, Cheng Wang, Xiaoyu Qin, Xiaosong Chen, Jing Chen, Zhenbing Chen, Tongkai Chen

**Affiliations:** a Department of Plastic Surgery and Regenerative Medicine, Fujian Medical University Union Hospital, Fuzhou, 350001, China; b Science and Technology Innovation Center, Guangzhou University of Chinese Medicine, Guangzhou, 510405, China; c Department of Hand Surgery, Union Hospital, Tongji Medical College, Huazhong University of Science and Technology, Wuhan, 430022, China; d Department of Dermatology, Wuhan No. 1 Hospital, Wuhan, 430022, China

**Keywords:** Diabetic wound, Mitochondrial dysfunction, Inflammation, Oxidative stress, Herbal self-assembled nanoparticles

## Abstract

The persistence of inflammation and mitochondrial dysfunction poses considerable challenges in the process of diabetic wound healing. Evidence from earlier research has revealed the significant activation of inflammation-related pathways in diabetic wound tissues. Thus, controlling inflammation may be the key to resolving non-healing diabetic wounds. Natural product-derived naringenin (Nar) and curcumin (Cur) can synergistically exert anti-inflammatory effects and accelerate mitochondrial repair. These compounds can promote the repair of damaged cells by improving mitochondrial function (restoring membrane potential, alleviating calcium overload, and inhibiting mitochondrial reactive oxygen species [ROS] production). Furthermore, they can activate the Nrf2/HO-1 pathway to enhance endogenous antioxidant defenses (SOD and CAT) and upregulate anti-inflammatory pathways, thus effectively suppressing NF-κB-mediated inflammatory cascades. In this study, we constructed a self-assembled herbal nanoparticle delivery system (NC NPs) composed of Nar and Cur. To enhance the applicability of this system for skin wounds, we prepared NC@Gel, a biocompatible thermo-sensitive hydrogel loaded with the NC NPs. *In vivo* experiments confirmed that NC@Gel not only provided synergistic anti-inflammatory and antioxidant effects but also regulated the phenotypic transition of macrophages, inhibits the secretion of pro-inflammatory factors, and ultimately promotes tissue regeneration in diabetic wounds. These findings shed light on the molecular mechanism through which NC@Gel exerts therapeutic effects via the Nrf2/NF-κB/mitochondrial functional axis. Overall, this study provides a novel nanomedicine-based strategy that could be translated for the clinical treatment of diabetic chronic wounds.

## Highlights

• NC@Gel synergistically exerted anti-inflammatory effects and accelerated mitochondrial repair.
• NC@Gel activated the Nrf2/HO-1 pathway to enhance endogenous antioxidant defenses and upregulated anti-inflammatory pathways.
• NC@Gel regulated the phenotypic transition of macrophages, and ultimately promoted tissue regeneration in diabetic wounds.

## Graphical abstract

The NC@Gel nanosystem is prepared via the self-assembly of the natural small molecules naringenin and curcumin into nanoparticles and the loading of these nanoparticles into a biocompatible hydrogel. This system effectively limits inflammation in diabetic wounds through three primary mechanisms: immune suppression, reactive oxygen species (ROS) scavenging, and mitochondrial repair.

**Figure:**
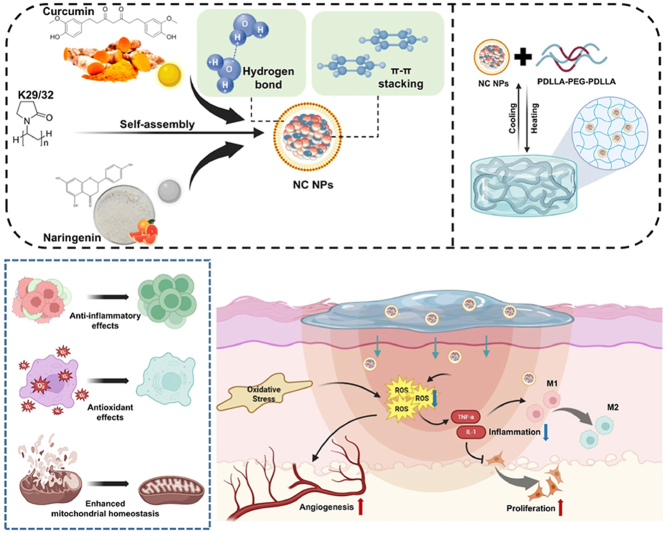

## 1 Introduction

Chronic non-healing wounds are common complication of diabetes [1]. Under normal conditions, wound healing typically occurs in four stages: hemostasis, inflammation, cell proliferation, and tissue remodeling [2]. However, in diabetic wounds, the dysregulation of the immune system leads to the generation of a persistent inflammatory environment characterized by hypoxia and hyperglycemia, including excessive reactive oxygen species (ROS) generation in mitochondria. Evidence shows that mitochondrial ROS (mtROS) can regulate immune responses and autophagy-related signaling pathways [3]. As central metabolic hubs, mitochondria not only regulate cellular metabolic states and metabolic crosstalk but also contribute to homeostasis through ROS-mediated signaling [4]. After the onset of diabetes, factors such as inflammation, oxidative stress, and ROS can all cause tissue damage and impede wound healing [5]. Therefore, breaking this vicious “inflammation-mitochondrial damage-ROS production” cycle is critical for accelerating diabetic wound repair.

Flavonoids, which are abundant in herbs, exhibit various pharmacological activities, including antioxidant and anti-apoptotic properties [6,7]. Naringin (Nar) is a natural flavonoid glycoside that exhibits potential in treating diabetes and its complications due to its diverse biological activities, particularly its antioxidant effects [8]. In addition, Nar can also inhibit nuclear factor kappa-B (NF-κB) phosphorylation to reduce endothelial cell apoptosis [9]. While restoring mitochondrial Ca^2+^ balance and hindering ROS production to attenuate endothelial cell damage [10]. Meanwhile, the non-polar polyphenol Curcumin (Cur) demonstrates multiple pharmacological activities and is an excellent anti-inflammatory agent [11]. Cur effectively promotes collagen biosynthesis and enhances fibroblast migration [12]. Moreover, Cur exhibits unique immunomodulatory functions by selectively inhibiting the activation of M1-type macrophages and downregulating pro-inflammatory mediators [13]. These findings collectively suggest that Nar and Cur may enhance diabetic wound healing in a synergistic manner [14]. However, the application of these compounds is limited by their poor stability, low bioavailability, and rapid metabolism [15,16].

Building on the identification of supramolecular self-assembled architectures, researchers have uncovered a diverse array of bioactive constituents derived from Chinese herbal medicines—including flavonoids, polyphenols, alkaloids, terpenoids, and proteins—that spontaneously organize into functional nanostructures through non-covalent molecular interactions [17,18]. The self-assembly of mono-molecular, bi-molecular, and multi-molecular systems has garnered growing interest for its capacity to enhance the therapeutic utility of natural compounds. Moreover, self-assembled systems that consist of natural small molecules offer distinct advantages, including high drug-loading capacity, inherent biocompatibility, reduced systemic toxicity, and the potential for synergistic pharmacological effects. As a result, these systems have emerged as promising candidates for next-generation nanomedicines [19]. However, free nanoparticles cannot effectively cover wounds and fail to achieve adequate exudate management and hemostasis. Thus, to prepare wound dressings, these nanoparticles can be incorporated into biocompatible thermosensitive hydrogels that adapt to wounds of different shapes and sizes [20].

**Fig. 1:**
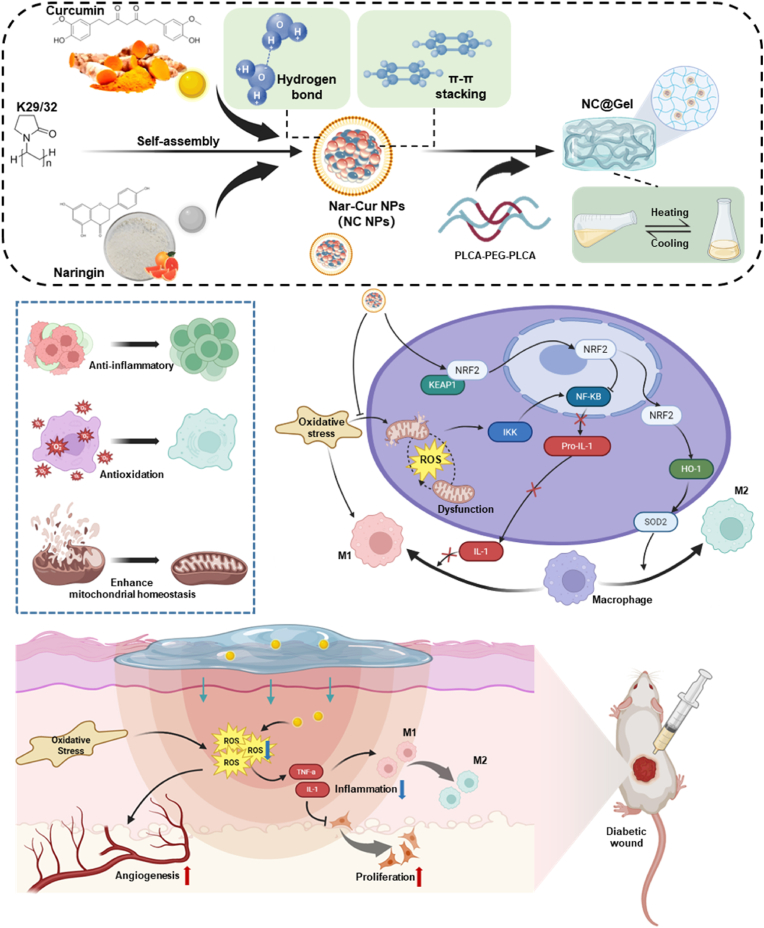
Schematic depicting the mechanisms of how NC NPs promote diabetic wound repair. Naringenin and curcumin could undergo self-assembly to form NC NPs, which were subsequently incorporated into a PDLLA-PEG-PDLLA matrix to form NC@Gel. The synthesized NC NPs exerted anti-inflammatory and antioxidant effects and enhanced mitochondrial function of HUVECs by regulating nuclear factor-erythroid 2-related factor 2 (Nrf2) and nuclear factor kappa-B (NF-κB) signaling. *In vivo* experiments confirmed that NC@Gel inhibits inflammation, scavenges reactive oxygen species (ROS), promotes vascular neogenesis, and induces anti-inflammatory macrophage polarization, thus accelerating the repair of diabetic wounds.

In this study, Nar and Cur were self-assembled into NC NPs via intermolecular interactions, such as π–π stacking and hydrogen bonding. After the self-assembly into nanoparticles, the biostability of Nar and Cur was significantly improved, their bioactivity was enhanced, and their biosafety was maintained. Subsequently, an injectable NC NPs-based hydrogel system (NC@Gel) was developed by incorporating NC NPs within a thermosensitive poly (D, L-lactide)-b-poly (ethylene glycol)-b-poly (D, L-lactide) (PDLLA-PEG-PDLLA) hydrogel. Our findings showed these NC NPs effectively scavenge ROS, reduce inflammation, and restore mitochondrial homeostasis. Our *in vivo* experiments further demonstrated that the NC@Gel not only exhibit strong anti-inflammatory effects but also promote macrophage reprogramming, ultimately accelerating diabetic wound healing (Fig. 1).

## 2 Materials and methods

### 2.1 Materials

Curcumin (Cur; ≥98 % purity) and naringenin (Nar; ≥98 % purity) were procured from Nantong Feiyu Biological Technology Co., Ltd. (Jiangsu, China). Dimethyl sulfoxide (DMSO) was purchased from Sigma-Aldrich (St. Louis, MO, USA), and povidone K29/32 (PVP) was bought from Ashland (Kentucky, USA). PDLLA-PEG-PDLLA (PEG, MW 2000 Da; PDLLA, MW 2000 Da) was purchased from the Jinan Daigang Biomaterial Co., Ltd. (Shandong, China).

Human umbilical vein endothelial cells (HUVECs; #GDC166) were purchased from the China Center of Type Culture Collection (CCTCC, Wuhan, China) and cultured according to the supplier's directions. Antibodies against CD31 (Cluster of differentiation 31, ab28364), SOD2 (ab68155), and COX IV (ab202554) were purchased from Abcam (Waltham, MA, USA). Antibodies against α-SMA (α-smooth muscle actin, 67735-1-Ig), CD86 (Cluster of differentiation 86, 26903-1-AP), CD206 (Cluster of differentiation 206, 81525-1-AP), Nrf2 (Nuclear factor-erythroid 2-related factor 2, 80593-1), and IL-10 (Interleukin-10, 60269-1-Ig) were purchased from Proteintech (Wuhan, China). Antibodies against NF-κB (Nuclear factor kappa-B, 8242T) was purchased from Cell Signaling Technology (Danvers, MA, USA). The Rhod-2 probe was provided by Yeasen Biotechnology (Shanghai, China). Mitochondrial superoxide indicator (mitoSOX) and mitochondrial membrane potential assay kit with JC-1 were purchased from Beyotime (Shanghai, China).

### 2.2 Data preprocessing and DEG identification

Microarray gene expression profiles related to diabetic foot ulcer (DFU) were retrieved from the Gene Expression Omnibus (GEO; https://www.ncbi.nlm.nih.gov/geo/). The dataset GSE80149 was selected for subsequent analysis. Batch effects were removed using the “sva” package in R (version 4.1.2; http://r-project.org/). Removal efficacy was validated using box plots, and inter-sample correction was assessed via a two-dimensional principal component analysis (PCA) cluster plot.

### 2.3 Screening and analysis of differentially expressed genes (DEGs)

The normalized and merged gene expression matrix was analyzed using the R package “limma” to identify significant DEGs, defined as genes with p < 0.05 and |fold change| > 2.0. Heatmaps were generated using the “pheatmap” package (https://CRAN.R-project.org/package=pheatmap), and volcano plots were constructed using the OmicStudio tool (https://www.omicstudio.cn/tool).

### 2.4 Functional enrichment analysis

Gene Ontology (GO), Kyoto Encyclopedia of Genes and Genomes (KEGG), and Gene Set Enrichment Analysis (GSEA) were performed on DEGs using the R package “clusterProfiler”. Results were visualized with “GOplot” and “enrichplot”. Significant terms were defined as those with false discovery rate (FDR) < 0.05 and p < 0.05.

### 2.5 Protein-protein interaction (PPI) network construction and hub gene analysis

A PPI network for DEGs was constructed using the STRING database (version 12.0; https://www.string-db.org), retaining interactions with the combined score >0.9. The network was visualized in Cytoscape (v3.9.1). Hub genes were identified by selecting the top 100 nodes from each algorithm-based ranking, and results were integrated using an UpSet plot generated with the R package “UpSetR”.

### 2.6 Preparation and characterization of NC NPs

NC NPs were prepared using stock solutions of Cur in DMSO (40 mg/mL; 6 mg), PVP in water (4 mg/mL; 12 mg), and Nar in DMSO (20 mg/mL; 6 mg). First, 12 mg of PVP was added to 19.4 mL pure water. Separately, the Cur and Nar stock solutions (6 mg, 150 μL each) were mixed evenly for 5 min via ultrasonication. The resulting Cur-Nar mixture was added dropwise to the PVP solution while stirring continuously at 1200 rpm at room temperature. Finally, the mixture was stirred for an additional 6 h until it became clear and transparent. The mixture was ultrasonicated in an ice bath for 15 min and stored at 4 °C.

An aqueous suspension of NC NPs was irradiated with a red laser, and the Tyndall effect was recorded with a camera. The particle size distribution, zeta potential, and polydispersity index (PDI) were recorded using dynamic light scattering (DLS) analysis (Nicomp Nano Z3000 instrument, Particle Sizing Systems, CA, USA) to assess the stability of the NC NPs. The NC NPs were morphologically characterized using scanning electron microscopy (SEM) and transmission electron microscopy (TEM). For Fourier transform infrared (FTIR) spectroscopy, all samples were mixed with KBr to produce tablets. Meanwhile, X-ray diffraction (XRD) analysis and thermogravimetry–differential scanning calorimetry (TG-DSC) were conducted to examine the crystallinity and thermal properties of different samples, respectively. Finally, the elements C, N, and O in the lyophilized NC NPs and physical mixtures was observed via X-ray photoelectron spectroscopy (XPS).

### 2.7 Preparation and characterization of NC@Gel

To prepare NC@Gel, 500 mg PDLLA-PEG-PDLLA was dissolved in a 2 mL NC NPs suspension (40 μg/mL); this mixture was shaken at 4 °C using a vertical shaker until it became completely clear.

The structure of NC@Gel was analyzed using SEM. At pre-determined temperature intervals, the samples were equilibrated for 15 min, and the vials were placed at an angle to detect gel flow. Sample rheology was assessed using a dynamic stress-controlled rheometer. Cold samples were added to parallel plates (20 mm in diameter) under initial temperature conditions of 25 °C. The storage and loss moduli (G′ and G″) were detected while the samples were heated from 20 °C to 45 °C (0.5 °C/min). Amplitude sweep (oscillation frequency, 1 Hz; strain range, 0.01–100 %) and frequency sweep (strain, 1 %; oscillation frequency, 0.1–100 rad s^−1^) tests were conducted. To examine shear viscosity, the shear rate was enhanced from 0.1 to 100 s^−1^ and then reduced from 100 s^−1^ to 0.1 s^−1^ at 25 °C.

### 2.8 Hemolysis assay

Hemolysis assays were performed to measure the effects of different biomaterials on red blood cells (RBCs) and to examine the biocompatibility of these biomaterials. In the first step, RBCs were obtained via centrifugation (3500 rpm, 10 min, and 4 °C) and then washed thrice with PBS (pH 7.4) until the supernatant became clear. Subsequently, the RBC suspension (200 μL) was diluted to 10 % (v/v) and mixed with 800 μL of water (negative control), PBS (positive control), or NC NPs (5, 10, 15, and 20 μg/mL) (experimental groups). All samples were incubated at 37 °C for 3 h and subsequently centrifuged (3500 rpm, 10 min, and 4 °C). Finally, the absorbance of the obtained supernatants was measured at 540 nm with a microplate reader.

### 2.9 Cell function assays

The Calcein AM/PI Live and Dead Cell Dual Staining Kit (Yeasen Biotechnology, Shanghai) utilizes dual Calcein AM and PI dyes to label live and dead cells, facilitating the assessment of cell viability and cytotoxicity. After staining, live cells can be observed under excitation at 490 nm, while dead cells become visible upon excitation at 545 nm.

Intracellular ROS levels were detected with a ROS Detection Kit (2,7-dichlorofluorescein diacetate, DCFH-DA, Nanjing Institute of Bioengineering, China) based on the kit instructions. Specifically, cells were incubated with DCFH-DA (30 min) after treatment with various biomaterials. Subsequently, cells were observed under a fluorescence microscope.

For cell proliferation analysis, 5-ethynyl-2′-deoxyuridine (EdU) staining was conducted (Beyotime, China). HUVECs were cultured in a 96-well tissue culture plate for 24 h and subjected to different treatments. They were then cultured further for another 24 h before staining and imaging.

Cell migration was assessed through scratch assays. A marker pen was used to draw a line on each cell culture plate (Corning, USA), which was subsequently seeded with cells. After the cell confluence reached 95–100 %, a scratch was made along this line using a 1 mL pipette tip. Floating cells were removed via PBS washes, and the remaining cells were treated with NC NPs. Images were obtained immediately after scratch induction. Then, after 24 h of incubation, crystal violet staining was performed, and additional images were obtained for documentation.

### 2.10 Detection of intracellular antioxidant activity

To measure superoxide dismutase (SOD) activity, the Total SOD Activity Detection Kit (NBT method, Beyotime, China) was employed. HUVECs were collected and subjected to lysis. Then, a supernatant was obtained by centrifuging the lysate. An NBT working solution and reaction starter solution were prepared. Meanwhile, a detection system was established based on the manufacturer's instructions, and samples were incubated at 37 °C (30 min) before absorbance measurements at 560 nm.

Malondialdehyde (MDA) levels in test samples were quantitatively detected using a Lipid Oxidation (MDA) Detection Kit (Beyotime, China). After lysing the cells and collecting the supernatant via centrifugation, a portion of the supernatant was used for protein quantification. The working solution was prepared per kit instructions, and a detection system was set up. The mixture was heated at 100 °C for 15 min, cooled, and centrifuged again. Finally, absorbance measurements were recorded at 532 nm and 600 nm to calculate the MDA content.

Catalase (CAT) activity was assessed using a Catalase Detection Kit (Beyotime, China). Following cell lysis, the cell supernatant was obtained via centrifugation, and a portion of this supernatant was used for protein content measurement. The working solution was prepared according to kit instructions, and the detection system was established. The sample was incubated at 25 °C for at least 15 min, and absorbance was measured at 520 nm to calculate the CAT activity of each sample.

### 2.11 Analysis of mitochondrial activity

The JC-1 probe was employed to measure mitochondrial membrane potential (Δψm). Subconfluent HUVECs were pretreated with NC NPs (0, 5, 10, and 20 μg/mL) for 12 h. This was followed by H_2_O_2_ (450 μM) treatment and JC-1 staining (37 °C, 20 min). Finally, images were obtained via confocal laser scanning microscopy (CLSM, Nikon).

Meanwhile, changes in mitochondrial calcium levels were measured using the Rhod-2/AM cell-permeable calcium ion probe. Subconfluent cells were pretreated with the same concentrations of NC NPs for 12 h. This was followed by treatment with H_2_O_2_ and two washes with D-Hanks. The cells were stained with Rhod-2/AM (37 °C, 30 min) and incubated with DMEM for 20 min following two more D-Hanks washes.

### 2.12 Ethics statement

All experiments involving animals were conducted according to the ethical policies and procedures approved by the Institutional Animal Care and Use Committee, Tongji Medical College, Huazhong University of Science and Technology, China (Approval no. 2021-S2789).

### 2.13 Establishment of the diabetic wound model

All animal experiments were approved by the Animal Care Committee of Tongji Medical College (Approval number: 2021-S2789). Male C57BL/6 mice (6–8 weeks, 20–25 g) were used in the study. Type I diabetes was induced by intraperitoneal injection of 50 mg/kg streptozotocin for 5 consecutive days, and blood glucose was monitored for 2 weeks thereafter. All experiments were conducted 4 weeks after hyperglycemia (glucose level >16.7 mmol/L) was detected. The mice were randomized into four groups of eight mice each: control group, diabetic group, diabetic + hydrogel group, and diabetic + NC@Gel group. Under sterile conditions, standardized full-thickness skin wounds (diameter = 10 mm) were induced on the midline of the backs of the mice using a biopsy puncher. The wounds of the mice were treated with a PBS solution, hydrogel, or NC@Gel. Subsequently, wound healing was monitored and analyzed using ImageJ.

### 2.14 Histological and immunofluorescence analysis

Wound tissues collected on day 14 were fixed, paraffin-embedded, and sectioned. The presence of granulation tissue and collagen deposition was assessed through hematoxylin and eosin (HE) and Masson's trichome staining, respectively. For immunofluorescence analysis, the sections were incubated with antibodies against CD31, α-SMA, CD86, CD206, IL10, and TNF-α (tumor necrosis factor-α) at 4 °C overnight. After three PBS washes, the sections were incubated with a secondary antibody (Aspen, China) at room temperature for 1 h. Meanwhile, the wound tissue samples harvested on day 4 were used to prepare cryosections and stained with dihydroethidium (DHE) to assess ROS levels. All sections were imaged using a microscope, and images were analyzed with ImageJ.

### 2.15 Statistics

Data, presented as the mean ± standard deviation (SD), were analyzed using GraphPad Prism software (version 9.0.2, La Jolla, CA, USA). Comparisons between two groups and among three or more groups were performed using an unpaired Student's t-test and one-way analysis of variance (ANOVA) with Tukey's post-hoc test, respectively. *P* < 0.05 was considered statistically significant.

## 3 Results

### 3.1 Delayed wound healing is caused by the diabetic inflammatory response

**Fig. 2:**
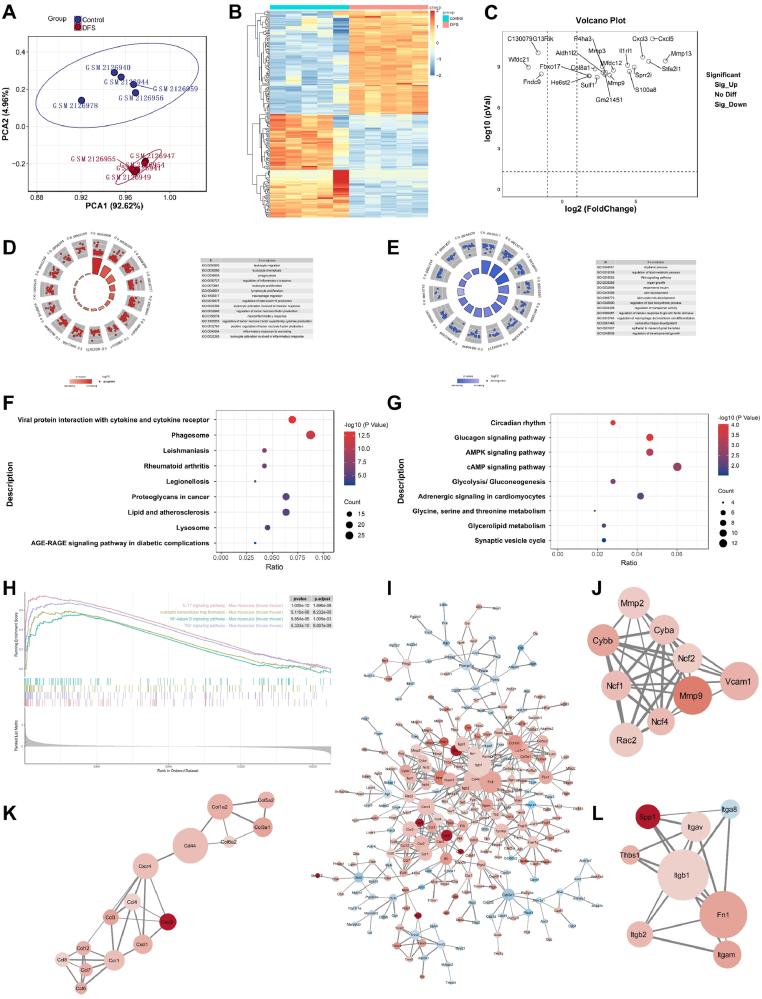
Inflammation is a major impediment limiting the healing process of diabetic wounds. (**A**) Batch-corrected principal component analysis (PCA) plot. (**B**) Heatmap of differentially expressed genes, with red and blue representing upregulated and downregulated differential genes, respectively. (**C**) Volcano plot of differentially expressed genes, with genes showing significant differences labelled in the plot. (**D & E**) GO analysis of upregulated genes (red) versus downregulated genes (blue) in diabetic foot ulcers. The top 10 GO items are listed in the upper plot, and the concentric circles in the nodes represent clustered differentially expressed genes. (**F & G**) KEGG pathway analysis of differential genes in diabetic foot ulcers. (**H**) GSEA of diabetic foot ulcers. (**I**) Protein interaction networks involved in diabetic foot ulcers and core sub-networks screened using the MCODE plugin. (**J-L**) Top three sub-networks. **Note:** Batch effect correction was performed for the GSE80149 dataset, and PCA was used to verify the correction effect. (For interpretation of the references to colour in this figure legend, the reader is referred to the Web version of this article.)

The delayed healing of diabetic wounds has been linked to the abnormal regulation of the inflammatory response. To understand the related molecular mechanisms, we screened a diabetic foot ulcer (DFU)-related microarray dataset (GSE80149) from the GEO database (Fig. 2A & S1). Screening criteria based on multiplicity of differences (|log2FC| > 2) and significance levels (p < 0.05) were applied (Fig. 2B and C). GO functional annotation indicated that the genes upregulated in DFU tissues are significantly enriched in biological processes such as inflammatory cell activation and migration, while the downregulated genes are mainly involved in tissue repair functions such as skin re-epithelialization, lipid metabolism regulation, and Wnt signaling. This suggests that immune and inflammatory responses act as key contributors to the delayed healing of DFU wounds (Fig. 2D and E).

Further KEGG pathway analysis demonstrated that the upregulated differential genes were significantly enriched in classical inflammatory pathways, such as the IL-17, TNF, and Toll-like receptor signaling pathways (Fig. 2F). Meanwhile, the downregulated genes were mainly involved in metabolic pathways closely related to wound healing, such as the AMPK, cAMP, and PI3K-Akt signaling pathways (Fig. 2G). In addition, GSEA further confirmed that IL-17 signaling, TNF signaling, NETosis, and NF-κB signaling play key regulatory roles in diabetic wound repair (Fig. 2H). Among them, the NF-κB signaling pathway, as a core molecular axis regulating the inflammatory response, shows significant activation under the pathological conditions of DFU, emerging as a critical candidate for subsequent studies on DFU management [21].

Finally, by constructing a protein interaction network, we found that CXCL2, MMP9, and SPP1 may play key roles in diabetic wounds (Fig. 2I). Evidence from the literature shows that MMPs can act as indicators of the inflammatory microenvironment in wound tissues. Elevated levels of MMP9 in diabetic wounds inhibit extracellular matrix synthesis and impede wound healing, and MMP9 upregulation is closely related to inflammation (Fig. 2J) [22]. Meanwhile, Cxcl2 is a chemokine with pro-inflammatory functions and regulates monocyte activation by influencing the recruitment of multinucleated cells (Fig. 2K) [23]. SPP1, an ECM protein and pro-inflammatory cytokine, modulates monocyte activation via the strong pro-inflammatory activation of monocytes, further exacerbating tissue inflammation (Fig. 2L) [24]. Together, these findings clearly demonstrate that inflammation is the primary problem during diabetic wound repair.

### 3.2 Preparation and characterization of NC NPs

**Fig. 3:**
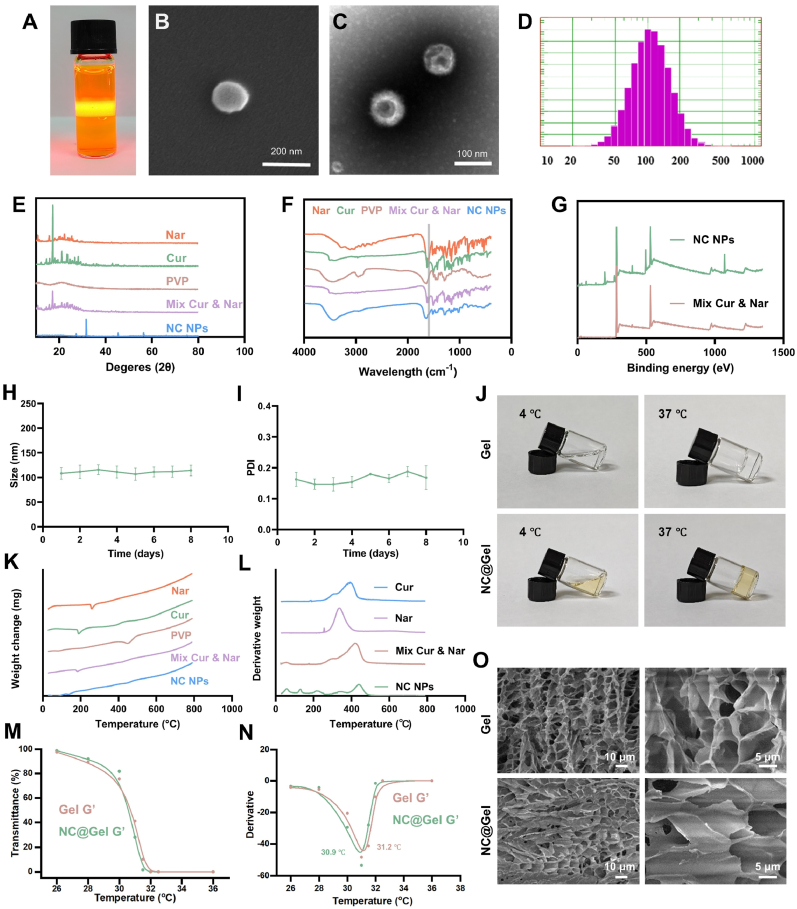
Characterization of NC NPs and NC@Gel. (**A**) The Tyndall effect exhibited by NC NPs. (**B**) SEM image of NC NPs. (**C**) TEM image of NC NPs. (**D**) Particle size analysis of NC NPs. (**E**) XRD scans of NC NPs. **(F**) FTIR spectroscopy analysis of NC NPs. (**G**) XPS spectra of NP NSs and a mixture of Cur and Nar. (**H**) Changes in the particle size of NC NPs. (**I**) PDI assay for NC NPs. (**J**) Transparency of the blank gel and NC@Gel at different temperatures. (**K**) Thermogravimetric analysis of NC NPs. (**L**) Heat flow analysis of NC NPs. (**M**) Estimation of hydrogel LCST based on temperature-dependent transparency differences. (**N**) Photographs of the blank hydrogel and NC@Gel at different temperatures. (**O**) Microstructure of the blank hydrogel and NC@Gel observed using SEM.

Nanosized drug delivery systems can improve the water solubility and bioavailability of hydrophobic drugs, including Cur and Nar. Here, Nar and Cur were directly self-assembled into nanoparticles (NC NPs) through π-π stacking and hydrogen bond interactions with hydrophilic PVP. This self-assembled nano-delivery system not only showed good biocompatibility but also improved the bioavailability and water solubility of Nar and Cur, allowing more efficient cellular uptake. As PVP was degraded, Nar and Cur were released, subsequently exerting a therapeutic effect. After screening, a 0.6 mg/mL aqueous solution of NC NPs containing 0.3 mg/mL Cur and 0.3 mg/mL Nar in PVP was found to be suitable for further experiments. The concentration of PVP in this solution was 0.6 mg/mL. Notably, the aqueous solution of NC NPs was transparent, and a light track could be observed upon laser beam irradiation (Fig. 3A). This light track was indicative of the presence of nanoparticles. Fig. 3B and C shows the SEM and TEM images of NC NPs. The NC NPs displayed negligible hemolytic activity (<4 %, Fig. S2), which demonstrated their good biocompatibility.

According to the results of DLS analysis, the average size of the NC NPs was ∼110 nm, and their PDI was 0.1, with zeta potential of −11.3 mV. Moreover, the NC NPs were found to be stable in solution for at least 11 days (Fig. 3D–H, & I). As shown in Fig. 3E, the XRD peaks of Cur and Nar completely disappeared in the XRD spectrum of NC NPs. These observations confirmed the intermolecular interactions among Cur, Nar, and PVP. In addition, they demonstrated that NP-entrapped Cur and Nar exist in an amorphous state. As shown in Fig. 3F, the FTIR spectrum of the physical mixture appeared as a combination of the physical spectra of Cur, Nar, and PVP. In contrast, in the FTIR spectrum of the NC NPs, some characteristic peaks of Cur and Nar were either shifted or absent, suggesting the presence of molecular interactions. FTIR peaks at 3100–3500 cm^–^^1^ typically correspond to O–H stretching vibrations. In the natural spectra of Cur, Nar, and PVP, the characteristic peaks related to hydrogen bonds appeared at 3416, 3111, and 3443 cm^−1^, respectively. Meanwhile, in the FTIR spectrum of NC NPs, the O–H stretching vibration peak shifted significantly and was detected at 3427 cm^−1^, indicating that hydrogen bonds were formed among Cur, Nar, and PVP. For PVP, the C–H stretching vibration appeared at 2956 cm^−1^. However, this peak was absent in the FTIR spectrum of NC NPs. Although the FTIR spectra of Cur and Nar contained a peak at 1628 cm^−1^, corresponding to the stretching vibration of the –C=C– group, no such peak was detected in the FTIR spectrum of NC NPs. Moreover, the stretching vibration of the –C=C– group (aromatic benzene ring) found in Cur and Nar was shifted from 1602 cm^−1^–1590 cm^−1^ in NC NPs. These differences reflected the intermolecular interactions among Cur, Nar, and PVP.

On XPS analysis, we found that NC NPs exhibited element absorption peaks similar to those observed in each of its individual components, further confirming the successful synthesis of the nanoparticles. Moreover, the TG-DSC analysis of Cur, Nar, and PVP; their physical mixture; and NC NPs yielded different derivative weight curves and heat flow curves (Fig. 3K and L). Compared to Cur, Nar, PVP, and their physical mixture, NC NPs showed a more gradual change in derivative weight, without any sudden weight fluctuations. In addition, compared with the individual components and their physical mixtures, the NC NPs exhibited no obvious heat absorption peak in the heat flow curves. The data thus indicated the absence of an obvious crystal melting process. These findings collectively indicated that NC NPs are amorphous in nature.

### 3.3 Characterization of NC@Gel

The temperature dependence of gel transparency was examined. The lower critical solution temperature (LCST) values of NC@Gel and the blank gel were 30.9 °C and 31.2 °C, respectively (Fig. 3M and N). Moreover, the temperature-induced gelation capacities of NC@Gel and the blank hydrogel were comparable (Fig. 3J), indicating that this property was unaffected by the introduction of NC NPs. Subsequently, SEM revealed that both the blank hydrogel and NC@Gel had homogenous pores, although the integration of NC NPs increased the pore size in NC@Gel (Fig. 3O).

In rheological tests, NC@Gel exhibited shear-thinning behavior (Fig. S3A). Specifically, its viscosity declined from 165.21 Pa to 37.65 Pa as the shear rate increased from 0.1 to 100 s^−1^, causing the gel to act like a viscous solution when shear force was applied. Owing to these characteristics, NC@Gel appeared to be suitable for injection into irregularly shaped wounds and for extrusion-based 3D printing applications. Strain amplitude sweep analyses (Fig. S3B) revealed that NC@Gel transitions from a gel state to a fluid state at 80 % applied strain. However, the blank hydrogel required strains greater than 100 % to achieve the same transition (gel state: G′ > G″ and flow state: G′ < G″). Finally, frequency sweep tests revealed the frequency dependence of the G′ and G″ moduli of both hydrogels. Both G′ and G″ increased as the angular frequency increased, with G′ consistently exceeding G″, demonstrating that a stable solid hydrogel was formed (Fig. S3C and D).

### 3.4 Biosafety of NC NPs

**Fig. 4:**
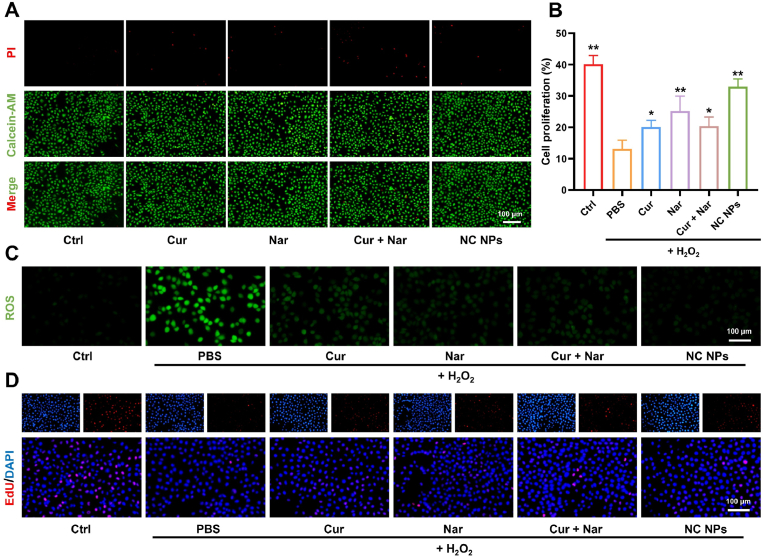
Enhanced effects of NC NPs when compared to individual components. (**A)** Comparison of biocompatibility between individual drugs (Cur and Nar), a simple mixture of components (Cur + Nar), and NC NPs. (**B & D)** Effect of each treatment agent on cell proliferation under oxidative stress (n = 5). (**C)** Comparison of the ROS-scavenging capacity of each treatment agent. ∗*p* < 0.05, ∗∗*p* < 0.01.

Biocompatibility is a key indicator for assessing the clinical potential of biomaterials. In the present study, to systematically evaluate the biosafety of NC NPs, we performed cytotoxicity assays using single-component materials, mixed systems, and NC NPs. Live-dead cell staining showed that none of the tested materials exhibited significant cytotoxicity within the tested concentration ranges (Fig. 4A). The cytotoxicity of NC NPs was further evaluated using the CCK-8 assay, which demonstrated no significant reduction in cell viability at concentrations of 0–50 μg/mL after 24 h of co-culture (Fig. S4A). However, extending the incubation to 48 h significantly decreased cell viability at higher NC NPs concentrations (>30 μg/mL) (Fig. S4B). This phenomenon could be attributed to the intracellular accumulation of nanoparticles at high treatment concentrations, leading to lysosomal overload. Based on these findings, a working concentration of NC NPs below 25 μg/mL was selected for subsequent experiments. To confirm the appropriateness of this NC NPs concentration, biosafety was further assessed via live-dead staining. Moreover, treatment with 20 μg/mL NC NPs maintained over 92 % cell viability for 48 h (Fig. S4C).

To investigate the specific mechanism of NC NP uptake by endothelial cells, blocking experiments were conducted using specific endocytosis inhibitors. Cells were pretreated with Filipin III (follicle-mediated endocytosis inhibitor), chlorpromazine (clathrin-mediated endocytosis inhibitor), amiloride (macropinocytosis inhibitor), and nystatin (lipid raft-mediated endocytosis inhibitor). As shown in Fig. S5, amiloride and nystatin significantly reduced the internalization efficiency of NC NPs. This indicated that macropinocytosis and lipid raft-mediated endocytosis are the primary pathways for NC NP uptake by endothelial cells.

### 3.5 Function-enhancing effects of NC NPs

Earlier studies have shown that Nar exhibits significant efficacy in regulating oxidative stress, inhibiting the progression of atherosclerosis, and preventing thrombosis. Notably, Nar can ameliorate ischemic injury by restoring ΔΨm via the modulation of the Bcl-2/Bax signaling pathway [25]. In addition, Cur has also demonstrated promising therapeutic potential for oxidative stress-related diseases owing to its excellent bioactive properties [26,27].

However, the effectiveness of both these compounds is generally limited by their low bioavailability. NC NPs, constructed in the present study via organic molecular self-assembly, not only appeared to enhance the solubility and stability of these compounds but also enabled the synergistic potentiation of their therapeutic effects.

To verify the enhanced functional performance of NC NPs, a cellular oxidative stress model was established using H_2_O_2_ treatment (Fig. 4B). Then, the ROS scavenging capacity of each treatment agent was examined. Nar and Cur, when used individually, reduced ROS levels by 61.28 % and 40.05 %, respectively. Meanwhile, the physical mixture of Nar and Cur further improved ROS scavenging to 87.13 %. In contrast, the self-assembled NC NPs exhibited the strongest ROS-scavenging capacity, reducing ROS levels by 92.7 %. Notably, the self-assembled NC NP group significantly outperformed both the single-component and physical mixture groups (Fig. 4C).

The proliferation-promoting effect of each component was also assessed using EdU staining. H_2_O_2_-induced oxidative stress markedly suppressed the proliferation of endothelial cells. Among the single components, Nar provided a 1.92-fold increase in the cell proliferation rate. However, NC NPs produced a more pronounced effect, yielding a 2.51-fold increase in cell proliferation (Fig. 4D). These findings indicated that the NC NPs produced through self-assembly not only retained the biological activity of their individual components but also showed significantly enhanced functions due to synergistic interactions.

### 3.6 Repair of oxidative stress-induced cellular damage by NC NPs

**Fig. 5:**
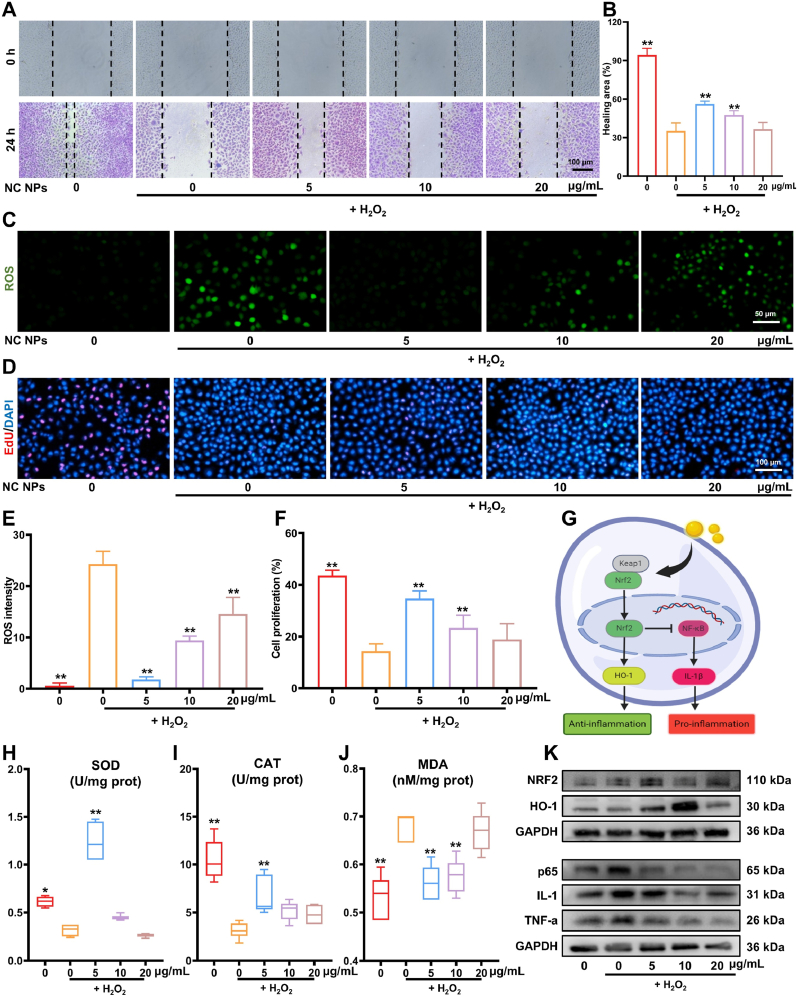
Ability of NC NPs to scavenge ROS efficiently. (**A & B**) NC NPs can promote endothelial cell migration (n = 5). (**C & E**) The ability of NC NPs to scavenge ROS is concentration-dependent, with better scavenging effects detected at lower concentrations (n = 5). (**D & F**) Under oxidative stress conditions, low concentrations of NC NPs exert strong pro-proliferative effects on endothelial cells (n = 5). (**G**) Schematic diagram depicting the anti-inflammatory effects of NC NPs. (**H-J**) Verification of the antioxidant performance of NC NPs based on SOD, MDA, and CAT levels (n = 5). (**K**) Intracellular protein levels in endothelial cells. ∗*p* < 0.05, ∗∗*p* < 0.01.

Oxidative stress is well recognized as a key pathological contributor to endothelial cell dysfunction during diabetic wound healing. Therefore, to systematically assess the effect of self-assembled NC NPs on oxidative stress-induced injury, we used H_2_O_2_ to establish a cellular oxidative stress model. In the present study, CCK-8 assays revealed that NC NPs could significantly increase the survival rate of endothelial cells under oxidative stress (Fig. S6). In addition, when the cell migration capacity was further assessed using scratch assays, the results revealed that oxidative stress significantly inhibited cell migration. Although these effects were significantly ameliorated by low concentrations of NC NPs (5–10 μg/mL), NC NP concentrations exceeding 20 μg/mL had a limited effect on promoting cell migration (Fig. 5A and B). This difference could be related to the increased metabolic burden in cells due to a high concentration of nanoparticles.

In terms of ROS scavenging capacity, oxidative stress led to an approximately 3.8-fold increase in intracellular ROS levels. Treatment with a low concentration of NC NPs (5 μg/mL) significantly reduced ROS levels (92.67 % reduction), while the ROS-scavenging effect of the high concentration group (20 μg/mL) was relatively weak (40.06 % reduction, Fig. 5C and E). The results of the EdU proliferation assay demonstrated that 5 μg/mL NC NPs could significantly promote cell proliferation (2.42-fold increase in cell proliferation rate), but this effect gradually weakened with increasing NC NP concentrations (Fig. 5D and F). This concentration-dependent effect could be related to the cellular uptake and metabolism of nanoparticles.

To elucidate the antioxidant mechanism of NC NPs, we assessed the activities of key antioxidant enzymes. Typically, intracellular SOD and CAT activities are often examined to understand the antioxidant capacity of cells. The results of the present study showed that treatment with 5 μg/mL NC NPs enhanced SOD activity by 3.9-fold and CAT activity by 2.19-fold. In contrast, the 20 μg/mL treatment group had a limited effect on the activities of both antioxidant enzymes (Fig. 5H and I). Furthermore, NC NPs significantly inhibited the production of the lipid peroxidation product MDA, and MDA levels were reduced by 82.34 % and 84.45 % in the 5 and 10 μg/mL NC NP groups. In contrast, no significant decrease in MDA levels could be noted in the 20 μg/mL NC NP group (Fig. 5J).

Molecular studies have shown that NC NPs retain the antioxidant properties of Nar and Cur, which are mediated by the Nrf2 pathway [28]. In the present study, qPCR and western blot analysis showed that 5–10 μg/mL NC NPs significantly upregulate Nrf2 and its downstream target gene HO-1 while inhibiting the expression of p65 (a key protein in the NF-κB pathway) and LC3 (autophagy marker) (Fig. 5G, K & S7). Both HO-1 and SOD are key antioxidant enzymes regulated by Nrf2 [29], and the activation of the NF-κB pathway induces the expression of pro-inflammatory factors such as TNF-α and IL-1β, which exacerbate oxidative damage [30]. Hence, these results suggest that NC NPs may exert their antioxidant and cytoprotective roles through the activation of the Nrf2 pathway and the inhibition of NF-κB signaling.

### 3.7 Amelioration of oxidative stress-induced damage by NC NPs via mitochondrial function modulation

**Fig. 6:**
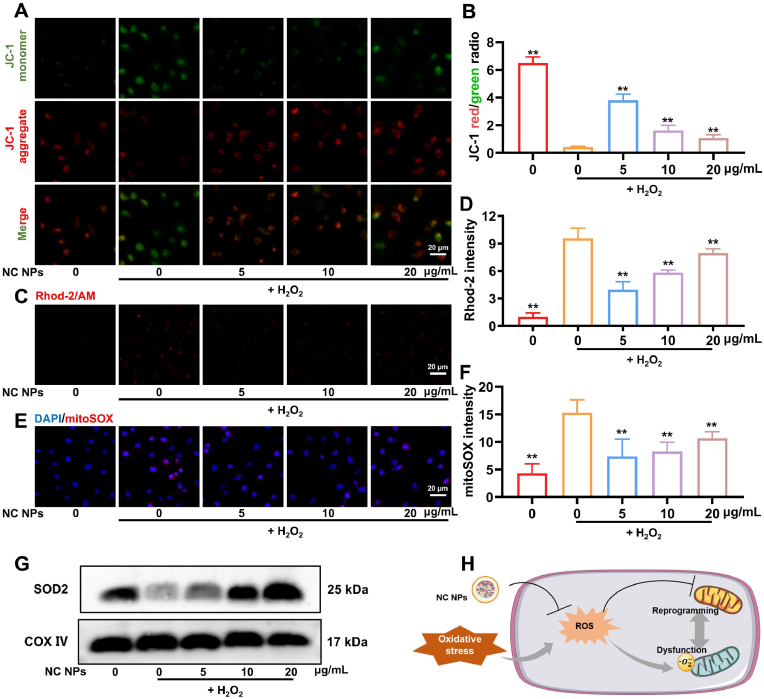
Ability of NC NPs to modulate mitochondrial function efficiently. (**A and B**) Pre-treatment of endothelial cells with NC NPs for 24 h, followed by the establishment of an oxidative stress model, to detect intracellular mitochondrial membrane potential (n = 5). (**C & D**) Rhod-2 reflected the intracellular Ca^2+^ content, and mitochondrial damage was assessed via Rhod-2 staining in each group (n = 5). (**E & F**) The mitoSOX dye could specifically target mitochondria and was used to assess mitochondrial superoxide anion production (n = 5). (**G**) Changes in the content of the antioxidant enzyme SOD2 within mitochondria. (**H**) Schematic representation of the mitochondrial function improvement induced by NC NPs. ∗*p* < 0.05, ∗∗*p* < 0.01.

In diabetes, the persistent hyperglycemic microenvironment causes the excessive accumulation of intracellular ROS. Mitochondria, as the metabolic center of the cell, are particularly vulnerable to such oxidative damage. Restoring mitochondrial homeostasis is thus important for reversing oxidative stress-related injury. In the present study, the protective effect of NC NPs on mitochondrial function was tested using a series of experiments. First, the changes in ΔΨm were assessed using JC-1 staining. While H_2_O_2_ treatment caused a significant decrease in intracellular ΔΨm, NC NP treatment restored ΔΨm. However, the improvements depended on the dose of NC NPs, with the best recovery detected in the 5 μg/mL NC NP group (58.68 % of the normal level) (Fig. 6A and B).

Mitochondrial calcium homeostasis is also disrupted under conditions of oxidative stress injury. Thus, we used the Rhod-2/AM fluorescent probe to detect mitochondrial calcium ion concentrations in endothelial cells. H_2_O_2_ treatment caused a 9.61-fold increase in mitochondrial Ca^2+^ levels. However, treatment with 5 μg/mL NC NPs significantly ameliorated calcium overloading (reduced by 58.60 %) (Fig. 6C and D). Under oxidative stress conditions, the mitochondrial content in cells significantly decreased. After low-dose NC NPs treatment, the intracellular mitochondrial content was markedly increased (2.53-fold increase) (Fig. S8). To further elucidate the role of NC NPs in the regulation of the redox state in mitochondria, we detected mtROS levels using the mitoSOX probe. Here, the results showed that oxidative stress resulted in an approximately 3.56-fold increase in mtROS levels in endothelial cells, but a low dose of NC NPs (5 μg/mL) significantly reduced mtROS content (by 51.69 %) (Fig. 6E and F). In addition, western blot analysis revealed that treatment with NC NPs increased the expression of the mitochondrial antioxidant enzyme SOD2 (Fig. 6G), although the increase differed at different treatment concentrations.

Based on these findings, we inferred that NC NPs improve mitochondrial function through multiple pathways (Fig. 6H): (1) attenuating oxidative stress-induced damage by scavenging mtROS; (2) regulating mitochondrial calcium homeostasis to maintain ionic homeostasis; and (3) enhancing the mitochondrial antioxidant defense system and increasing the expression of antioxidant enzymes such as SOD2.

### 3.8 NC@Gel hydrogel system for diabetic wound healing

Persistent oxidative stress in the microenvironment of diabetic wounds significantly impairs the wound healing process [2]. Based on our previous findings that demonstrated the potent ROS-scavenging capacity and mitochondrial homeostasis-regulating properties of NC NPs, we developed an injectable NC@Gel hydrogel system for diabetic wound treatment. This hydrogel exhibited excellent biocompatibility and shape adaptability, allowing it to conform precisely to wounds and skin defects of varying shapes and sizes.

**Fig. 7:**
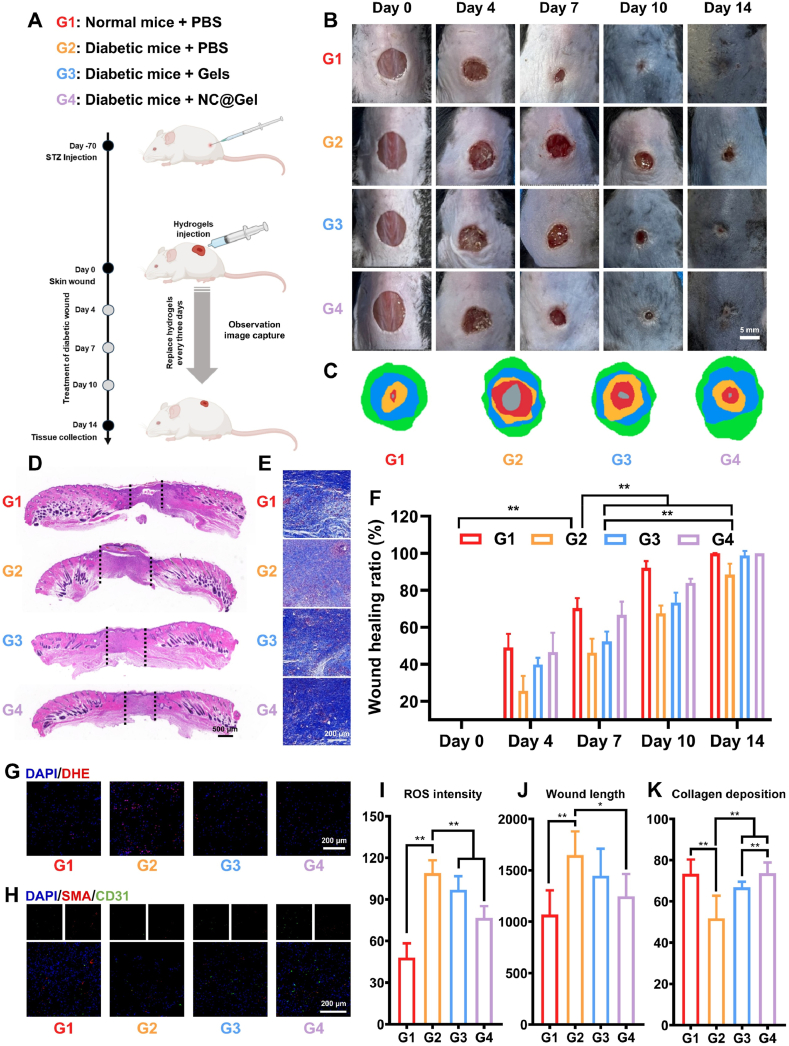
NC@Gel can promote diabetic wound repair. (**A**) Treatment strategy for diabetic wounds using NC@Gel. (**B & C**) Representative images showing wound healing at each stage: on the first day of treatment, fourth day of treatment, seventh day of treatment, tenth day of treatment, and fourteenth day of treatment. (**D**) Representative hematoxylin and eosin-stained sections from each group on the fourteenth day of treatment. (**E**) Representative Masson staining images. (**F**) Healing curves of each treatment group (n = 4). (**G**) Assessment of ROS content in the wound area based on DHE staining (n = 3). (**H**) Neovascularization in different groups of mice on the fourteenth day of treatment. (**I**) Quantification of ROS levels in wound tissues (n = 3). (**J**) Comparison of granulation tissue length (n = 4). (**K**) Comparison of collagen deposition (n = 4). ∗*p* < 0.05, ∗∗*p* < 0.01.

To evaluate *in vivo* therapeutic effects, a diabetic mouse model with full-thickness skin defects was established. The experimental animals were randomly assigned to four groups: control, diabetic (Dia) + PBS, Dia + Gel, and Dia + NC@Gel. Treatments were administered on postoperative days 0, 4, 7, and 10, and the outcomes were evaluated on day 14 (Fig. 7A). Wound healing was monitored over the experimental duration. The findings revealed that the NC@Gel treatment group exhibited a significantly faster healing rate than the other groups at all time points, with near-complete wound closure achieved by day 14 (Fig. 7B, C, & F). Notably, NC@Gel outperformed the single Gel treatment group, suggesting that the synergistic action of Nar and Cur in the NC NPs played a key role in enhancing therapeutic outcomes in diabetic wounds.

Histological analysis further confirmed the pro-healing effects of NC@Gel. By postoperative day 14, the length of the granulation tissue in the NC@Gel group was markedly increased, and the tissue displayed a more mature structural organization (Fig. 7D and J). In addition, Masson's trichome staining showed more extensive collagen deposition in the wound area in the NC@Gel group, with the fibers being arranged in a denser and more orderly pattern (Fig. 7E and K). To evaluate the *in vivo* antioxidant effects of NC@Gel, DHE staining was performed, and ROS levels in the wound area were measured on postoperative day 7. In this experiment, tissues from the diabetic wound group displayed strong red fluorescence, indicating elevated ROS levels. However, NC@Gel treatment significantly reduced ROS levels by approximately 29.62 %, providing a greater reduction than Gel treatment alone (11.03 %) (Fig. 7G and I). Finally, neovascularization was assessed using CD31 and α-SMA immunofluorescence staining. The NC@Gel group showed a substantial increase in the density of neovascularization, indicating enhanced angiogenesis and improved nutrient supply to support wound healing in this group (Fig. 7H).

### 3.9 Anti-inflammatory effects of NC@Gel mediated by the nrf2/NF-κb signaling pathway

**Fig. 8:**
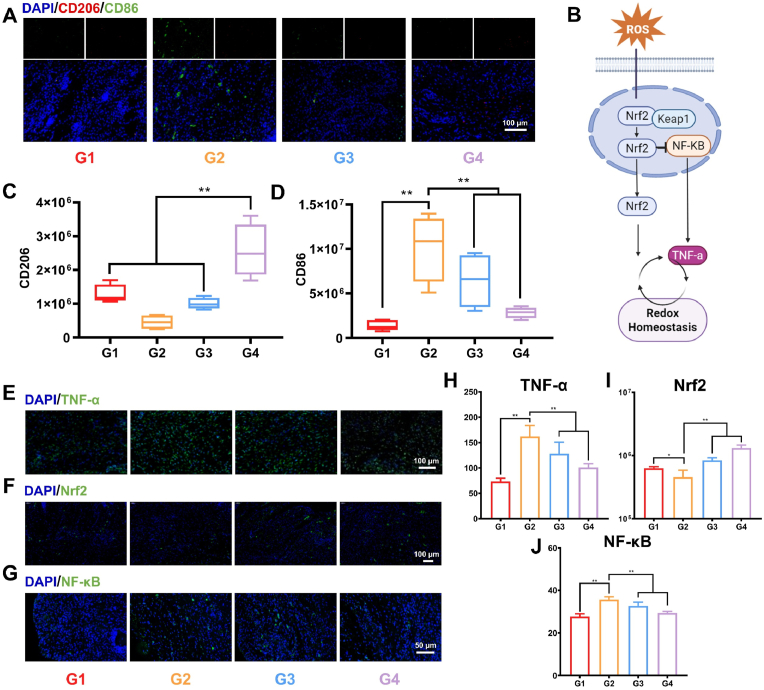
NC@Gel promotes wound repair via the modulation of ROS. (**A**) Immunofluorescence analysis of CD86 (M1 marker) and CD206 (M2 marker). (**B**) Schematic representation of the action of NC NPs. (**C**) Quantitative analysis of CD86 expression in wound tissues (n = 4). (**D**) Quantification of CD206 expression in wound tissues (n = 4). (**E-G**) Immunofluorescence analysis comparing the expression of TNF-α, Nrf2, and NF-κB in wound tissues. (**H-J**) Quantitative analysis of TNF-α, Nrf2, and NF-κB expression in wound tissues (n = 3). ∗*p* < 0.05, ∗∗*p* < 0.01.

Macrophage polarization is recognized to play a key regulatory role in the wound healing process. M1-type macrophages (CD86^+^) are primarily involved in promoting inflammation, while M2-type macrophages (CD206^+^) contribute to tissue repair and regeneration. In the present study, immunofluorescence staining on day 14 of treatment revealed that wounds in the diabetic group remained dominated by M1-type macrophages. In contrast, NC@Gel treatment significantly promoted macrophage polarization toward the M2 phenotype (Fig. 8A–D). Further analysis of inflammatory markers in the wound tissues showed that NC@Gel decreased the levels of the cytokine TNF-α (37.72 % reduction versus the diabetic group) (Fig. 8E and H). Meanwhile, NC@Gel increased the level of IL-10 (10.79 % higher than that of the diabetes group) (Fig. S9). These results indicated that NC@Gel exhibits strong immunomodulatory properties.

To elucidate the molecular mechanisms underlying the anti-inflammatory action of NC@Gel, we examined the levels of key regulators in the Nrf2 and NF-κB signaling pathways. Immunohistochemical analysis demonstrated that NC@Gel significantly upregulated Nrf2 expression in the wound tissue (∼1.44-fold increase) while simultaneously inhibiting the nuclear translocation of NF-κB p65 (∼17.7 % decrease) (Fig. 8F, G, I, & J). These findings supported the hypothesis that in diabetic conditions, chronic oxidative stress hinders wound healing by suppressing Nrf2 signaling pathway activity and activating NF-κB signaling, thereby disrupting the inflammatory microenvironment. NC@Gel effectively reversed this pathological process by scavenging ROS, activating the Nrf2 pathway, and suppressing NF-κB signaling pathway activation (Fig. 8B).

Finally, we assessed the *in vivo* biosafety of NC@Gel based on HE staining and toxicological analyses of major organs (heart, liver, spleen, lungs, and kidneys) after 14 days of treatment. These experiments revealed no noticeable abnormalities or pathological changes, confirming the good biocompatibility of NC@Gel (Fig. S10).

## 4 Discussion

Diabetic wounds are characterized by several pathological changes, primarily including a perturbed immune microenvironment, a persistence of excessive inflammatory responses, and an abnormal accumulation of ROS [31,32]. Among these, inflammatory responses may be the core pathological mechanism leading to the diabetic microenvironment. This persistent inflammatory state not only promotes the release of several inflammatory cytokines (e.g., TNF-α and IL-6) but also significantly inhibits the proliferation and migration of repair-associated cells, thus hindering the wound healing process. Chronic inflammatory stimulation induces the aberrant polarization of macrophage cells, leading to the over-activation of M1-type macrophages and the simultaneous inhibition of M2-type macrophages [33]. This imbalance in the state of macrophage polarization generates a vicious circle, causing the abnormal accumulation of ROS, which further exacerbates tissue injury through a positive feedback mechanism [34]. In the present study, further data analysis revealed that the NF-κB signaling axis potentially plays a key regulatory role in this pathological process. Therefore, its inhibition could effectively remediate the imbalance in macrophage polarization, attenuate the inflammatory response, and reduce the overall levels of oxidative stress. In addition, our protein interaction network analysis revealed that core proteins such as SPP1, CXCL2, and MMP9 are closely involved in the regulation of macrophage function in diabetic wounds. Therefore, therapeutic strategies for diabetic wounds should focus on i) controlling excessive inflammatory responses, ii) scavenging excessive ROS, and ii) shifting the state of macrophage polarization towards an anti-inflammatory state. The synergistic effects of these three key interventions could provide new therapeutic avenues for diabetic wound healing.

In recent years, the application of self-assembled nanoparticles to improve the effects of herbal medicines has attracted increased attention. The preparation of carrier-free nanomedicines via the direct self-assembly of active monomers can diversify existing herbal medicine formulations, improve their biostability and water solubility, and even contribute to physiological barrier penetration, tissue targeting, and functional coordination [35]. Beyond this, even limitations related to the solubility, immunogenicity, premature release, and adverse effects of herbal compounds can be easily addressed through the application of nanotechnology [36]. Self-assembly can be mediated by several interactive forces, including π-π interactions, charge-based interactions, hydrophobic interactions, hydrogen bonding, and van der Waals forces [37]. Although hydrogen bonding is used to generate a vast majority of current self-assembled nanoparticles, here, we leveraged a combination of π-π and hydrogen bonding interactions to enhance the mechanical properties and self-healing ability of prepared hydrogels.

The core pathological mechanisms underlying the non-healing of diabetic wounds include chronic hyperglycemia-induced oxidative stress, cellular inflammation, and mitochondrial dysfunction. All of these lead to a persistent inflammatory microenvironment and impaired tissue repair [38,39]. In addition, the hyperglycemic microenvironment damages mitochondria through mechanisms such as the polyol pathway, Advanced Glycation End-Products (AGEs), and the protein kinase C pathway, resulting in ROS overload. This induces cellular inflammatory secretion and triggers a positive feedback loop, leading to a prolonged inflammatory phase that delays the repair process [40].

Mitochondrial homeostasis is a key switch in the regulation of cellular inflammation. The release and accumulation of mitochondria-generated ROS (mtROS) in the cytoplasm induce intracellular oxidative stress and damage, which in turn disrupt cellular function by downregulating the expression of nitric oxide synthase (eNOS). In addition, mtROS also accelerates the accumulation of oxidized low-density lipoprotein (oxLDL), triggering subsequent apoptosis and inflammation [28]. Mitochondrial damage induces cellular inflammation by activating the NLRP3 signaling pathway, which triggers inflammasome activation and ROS overload [41]. When mitochondrial complex I is disrupted, glycolysis is upregulated, leading to increased mtROS production. These metabolic changes lead to a stronger inflammatory response in myeloid cells. The deletion of TFAM reduces mtDNA stability, disrupts mitochondrial biogenesis, increases mtROS production in renal tubular cells, and ultimately causes mitochondrial respiratory dysfunction and a cellular inflammatory response [42]. In contrast, enhancing mitochondrial autophagy via activation of the PINK1/Parkin pathway can reverse the inflammatory activation of macrophages caused by mitochondrial damage [43]. Collectively, these findings underscore the pivotal role of mitochondrial function in regulating intracellular oxidative stress and suppressing inflammatory responses, offering a potential therapeutic target for diabetic wound treatment.

Given the close connection between mitochondrial damage and the inflammatory response, we attempted to synthesize a bio-nanomaterial that enhances mitochondrial homeostasis while also scavenging ROS and mitigating inflammation to reduce cellular inflammation and accelerate wound healing. Nar not only inhibits the transcription of cytokine genes but also promotes the lysosomal degradation of cytokine proteins [7]. Owing to its antioxidant and anti-inflammatory properties, Nar reduces excessive ROS production by restoring mitochondrial Ca^2+^ homeostasis and upregulates the intrinsic antioxidant activity in endothelial cells, thereby alleviating lipid peroxidation injury [10,25]. Meanwhile, Cur possesses anti-inflammatory and antioxidant properties and is known to attenuate inflammatory responses by modulating mTOR to optimize lipolytic metabolism and inhibit autophagy [44,45]. Similarly, Cur can ameliorate mitochondrial damage and inhibit NLRP3 inflammasome activity and synovitis by inhibiting the NF-κB signaling pathway [46]. Therefore, the combination of Nar and Cur can target both upstream and downstream elements of the inflammatory signaling pathway, blocking the inflammatory cascade and enhancing anti-inflammatory efficacy. This synergistic approach allows for the administration of lower effective doses of each compound, reducing potential toxicity and achieving the goal of cost reduction and increased therapeutic efficacy.

The Nrf2 signaling pathway contributes to the maintenance of cellular redox homeostasis. Under oxidative stress, Nrf2 transcriptionally regulates ROS detoxification and antioxidant genes by translocating from the cytoplasm to the nucleus, where it binds to antioxidant response elements (AREs) [47,48]. Meanwhile, Cur supramolecular nanoparticles inhibit inflammation via the TLR4/NF-κB signaling pathway, mitigate oxidative damage by influencing Nrf2/HO-1 signaling, promote macrophage reprogramming, and accelerate the repair caused by ulcerative colitis [49]. NF-κB activation then induces the expression of pro-apoptotic genes, contributing to neuronal death. In addition, it also initiates an inflammatory cascade that drives pro-inflammatory cytokine production (e.g., TNF-α, IL-1β, and IL-6), which further worsen tissue damage through increased inflammation and immune cell infiltration [50]. Cur has been found to directly inhibit NF-κB activation, suppress microglial and astrocytic activity, downregulate TNF-α and IL-1β, and protect damaged neurons [51]. In the present study, we found that NC NPs could activate the Nrf2 pathway and enhance intracellular antioxidant capacity via the Nrf2/HO-1 axis. Moreover, these NPs also inhibited pro-inflammatory factor release and reduced oxidative stress by suppressing the NF-κB pathway.

Overall, our experiments demonstrated that the herbal nanoplatform composed of Nar and Cur effectively reduced persistent inflammation in the diabetic wound microenvironment by scavenging excessive intracellular ROS, improving mitochondrial function, and promoting macrophage reprogramming, ultimately accelerating diabetic wound repair. Notably, the specific mechanisms involved the activation of Nrf2 signaling and the inhibition of NF-κb signaling. Despite these strengths, our study has limitations that warrant attention. Firstly, we primarily focus on molecular mechanisms related to inflammation and oxidative stress, while the potential roles of NC NPs in other cellular processes, such as autophagy and pyroptosis, have not been thoroughly investigated. Secondly, long-term safety is still required for the clinical translation of NC@Gel.

## 5 Conclusion

In order to effectively disrupt the vicious cycle of “inflammation-mitochondrial damage-ROS production”, in this study, we developed an innovative herbal hydrogel based on Nar and Cur. NC@Gel efficiently scavenged intracellular ROS, significantly enhanced mitochondrial membrane stability, and improved cellular antioxidant capacity, thereby reversing inflammatory damage. In addition, further experiments revealed that the reparative effects of NC@Gel were primarily mediated through Nrf2 and NF-κB axis regulation. Finally, our *in vivo* experiments confirmed that NC@Gel not only provided synergistic anti-inflammatory and antioxidant effects but also promoted tissue regeneration in diabetic wounds. Thus, NC@Gel is a promising self-assembled nanomaterial for diabetic wound management. These results also demonstrated that NC@Gel could promote diabetic wound healing, and its easy-to-use nature underscores its potential as a practical clinical agent for treating diabetic wounds.

## CRediT authorship contribution statement

**Jiahe Guo:** Software, Methodology, Formal analysis, Conceptualization. **Ben Hu:** Writing – original draft, Investigation, Conceptualization. **Yi Wei:** Methodology. **Guopan Cheng:** Investigation. **Cheng Wang:** Conceptualization. **Xiaoyu Qin:** Writing – original draft. **Xiaosong Chen:** Project administration. **Jing Chen:** Methodology. **Zhenbing Chen:** Writing – original draft. **Tongkai Chen:** Writing – review & editing, Investigation, Funding acquisition, Conceptualization.

## Declaration of competing interest

The authors declare no conflict of interest.

## Data Availability

Data will be made available on request.
