# Supplementary material for: Hydrogel delivering self-assembled herbal nanoparticles accelerates diabetic wound healing through mitochondrial regulation
Source: Mater Today Bio. 2025 Oct 17;35:102417. doi: 10.1016/j.mtbio.2025.102417 (PMC12569843; doi:10.1016/j.mtbio.2025.102417)
Supplement: Multimedia component 1 [file mmc1.docx]

**Supporting Information**

**Hydrogel Delivering Self-Assembled Herbal Nanoparticles Accelerates Diabetic Wound Healing through Mitochondrial Regulation**

Jiahe Guo^1, 3, #^, Ben Hu^2, #^, Yi Wei^2, #^, Guopan Cheng^2^, Cheng Wang^3^, Xiaoyu Qin^2^, Xiaosong Chen^1,^ *, Jing Chen^4,^ *, Zhenbing Chen^3,^ *, Tongkai Chen^2,^ *

^1^ Department of Plastic Surgery and Regenerative Medicine, Fujian Medical University Union Hospital, Fuzhou 350001, China

^2^ Science and Technology Innovation Center, Guangzhou University of Chinese Medicine, Guangzhou 510405, China

^3^ Department of Hand Surgery, Union Hospital, Tongji Medical College, Huazhong University of Science and Technology, Wuhan 430022, China

^4^ Department of Dermatology, Wuhan No. 1 Hospital, Wuhan 430022, China

^#^ These authors contributed equally to this work.

* To whom correspondence should be addressed:

1. Tongkai Chen

Science and Technology Innovation Center, Guangzhou University of Chinese Medicine, 12 Jichang Road, Guangzhou 510405, China

Tel.: +86 20 36585707

E-mail: chentongkai@gzucm.edu.cn

2. Zhenbing Chen

Department of Hand Surgery, Union Hospital, Tongji Medical College, Huazhong University of Science and Technology, Wuhan 430022, China

E-mail: zbchen@hust.edu.cn

3. Jing Chen

Department of Dermatology, Wuhan No. 1 Hospital, Wuhan 430022, China

E-mail: cj727384211@gmail.com

4. Xiaosong Chen

Department of Plastic Surgery and Regenerative Medicine, Fujian Medical University Union Hospital, Fuzhou 350001, China

E-mail: chenxiaosong74@163.com

**
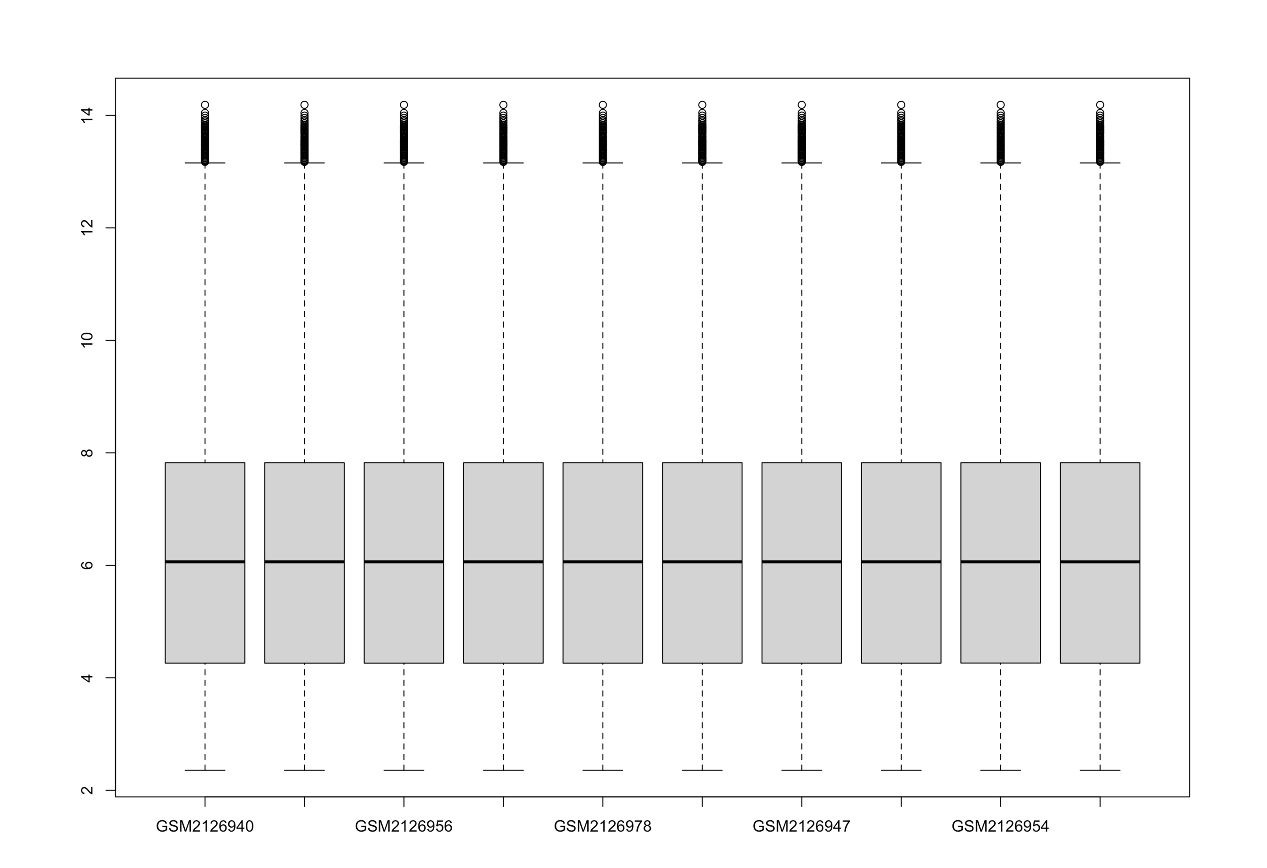
**

Figure S1 Standardization the GSE80419 dataset to remove batch effects.

**
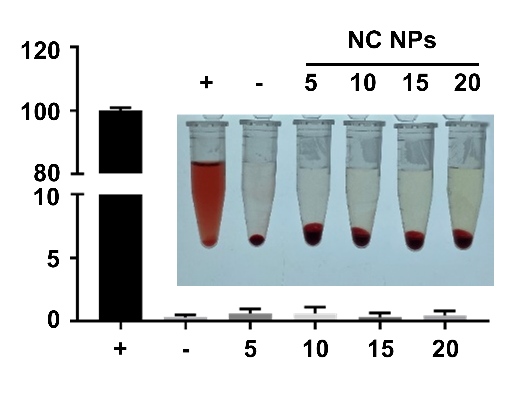
**

Figure S2 Hemolysis assay for NC NPs (n=6).

**
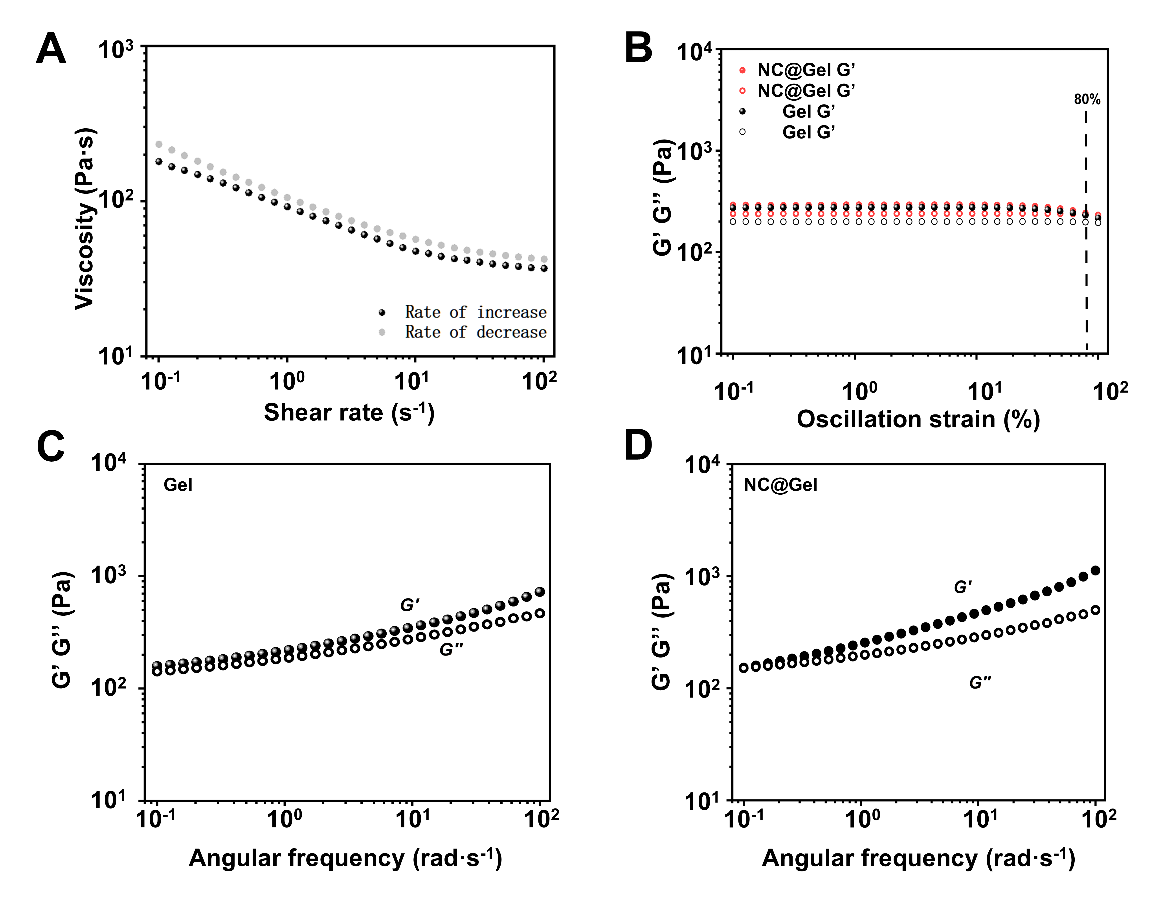
**

Figure S3 (A) Viscosity of NC@Gel at shear rates of 0.1 to 100 s^−1^. (B) Strain-dependent oscillatory shear rheology of the blank hydrogel and NC@Gel. (C) Frequency spectra of the G′ and G″ moduli of the blank hydrogel. (D) Frequency spectra of the G′ and G″ moduli of NC@Gel.

**
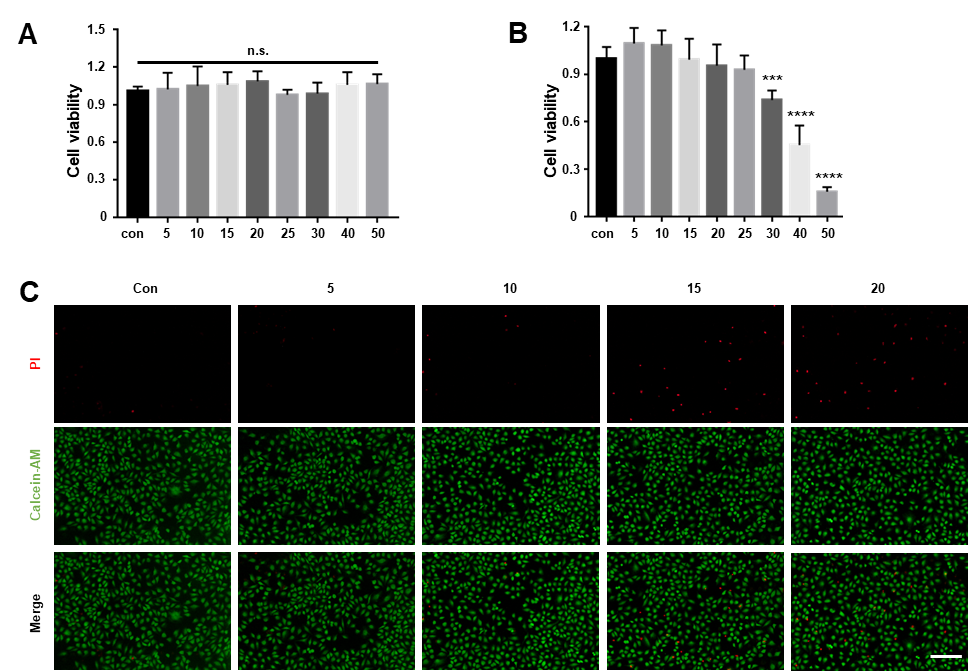
**

Figure S4 (A-B) Biocompatibility assay of different materials across various treatment concentrations after 24 h (A) and 48 h (B) of co-culture (n=5). (C) Live-dead cell staining to assess biosafety after 48 h of co-culture with different concentrations of NC NPs (n=5). Scale bar: 100 μm. *** *p*  < 0.001, **** *p*  < 0.0001.

**
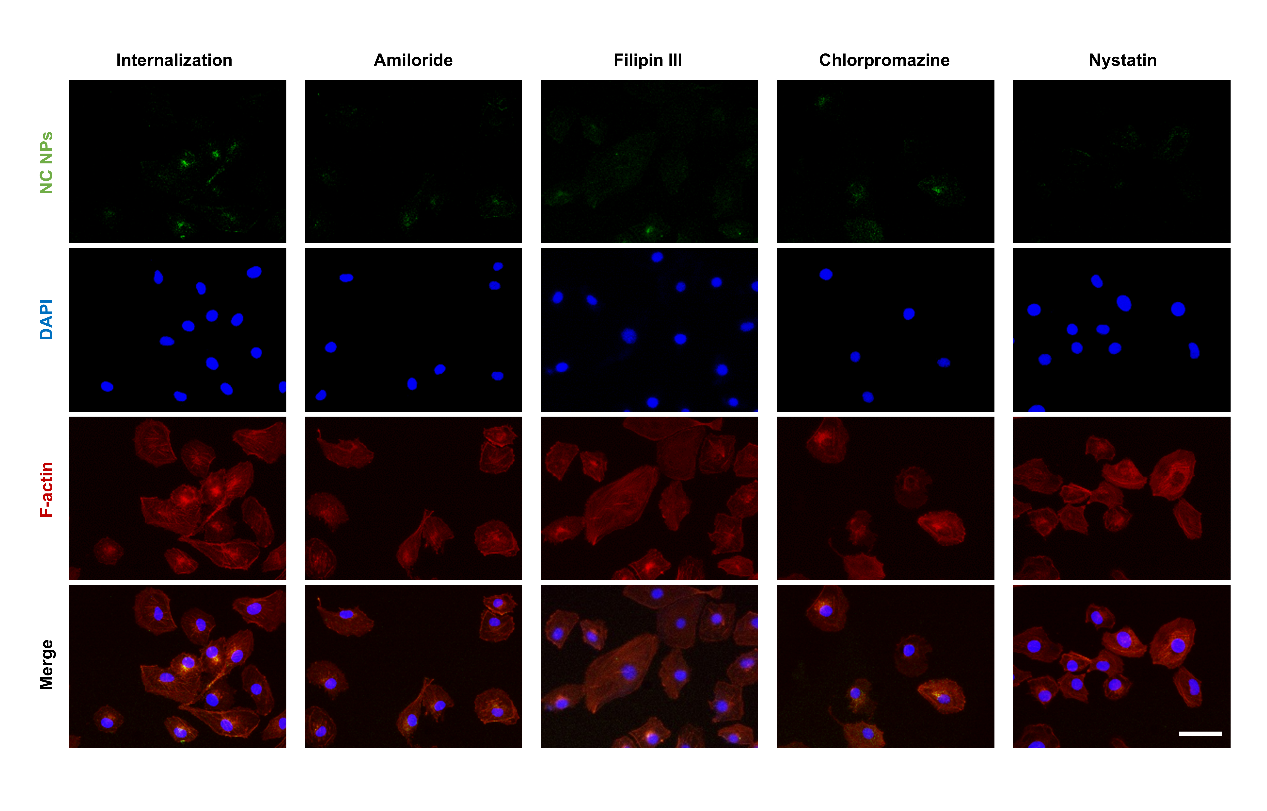
**

Figure S5 Analysis of the mode of NC NP uptake based on inhibitor treatment. Scale bar: 25μm.

**
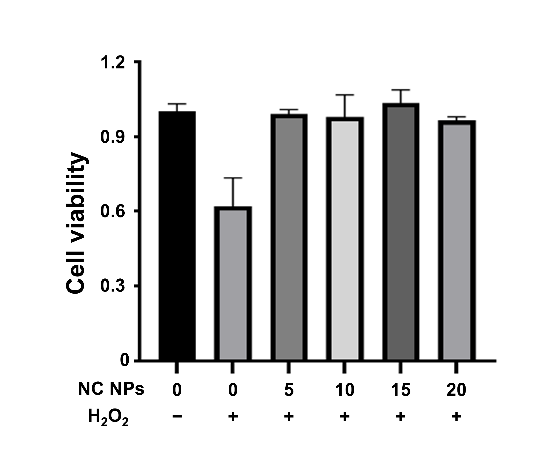
**

Figure S6 Cell activity assay after 24 h of NC NPs pre-treatment followed by H_2_O_2_ treatment (n=5).

**
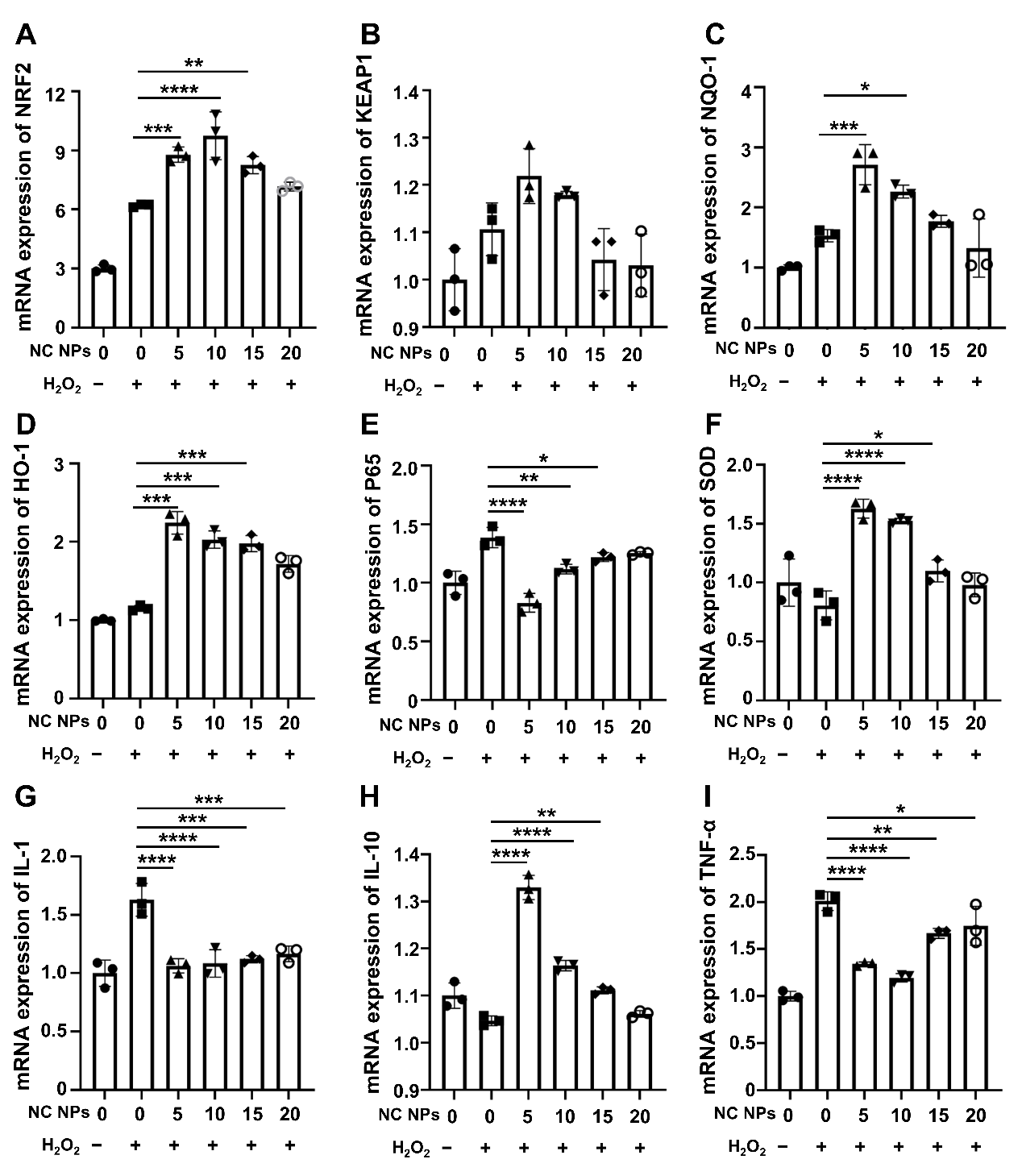
**

Figure S7 Expression of relevant genes detected after treatment with NC NPs using qRT-PCR (n=5). * *p*  < 0.05, ** *p*  < 0.01, *** *p* < 0.001, **** *p*  < 0.0001.


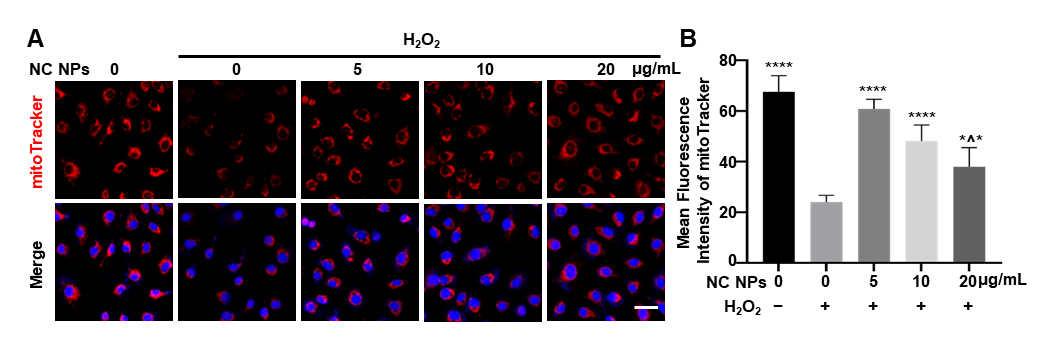


Figure S8 MitoTracker (Red) and DAPI (Blue) image (A) and MitoTracker fluorescence intensity (B) within cells (Scale bar: 20 μm; n=5). *** *p* < 0.001, **** *p* < 0.0001.


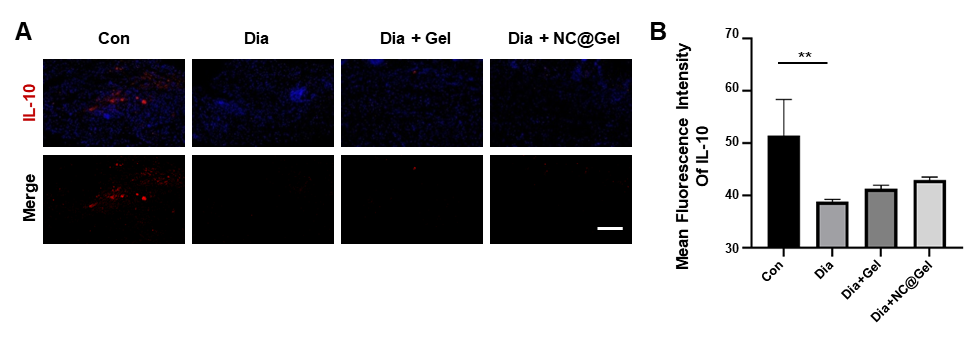


Figure S9. Immunofluorescence analysis (A) and fluorescence intensity (B) comparing the expression of IL-10 in wound tissues (Scale bar: 100 μm; n=3). ** *p* < 0.01.

**
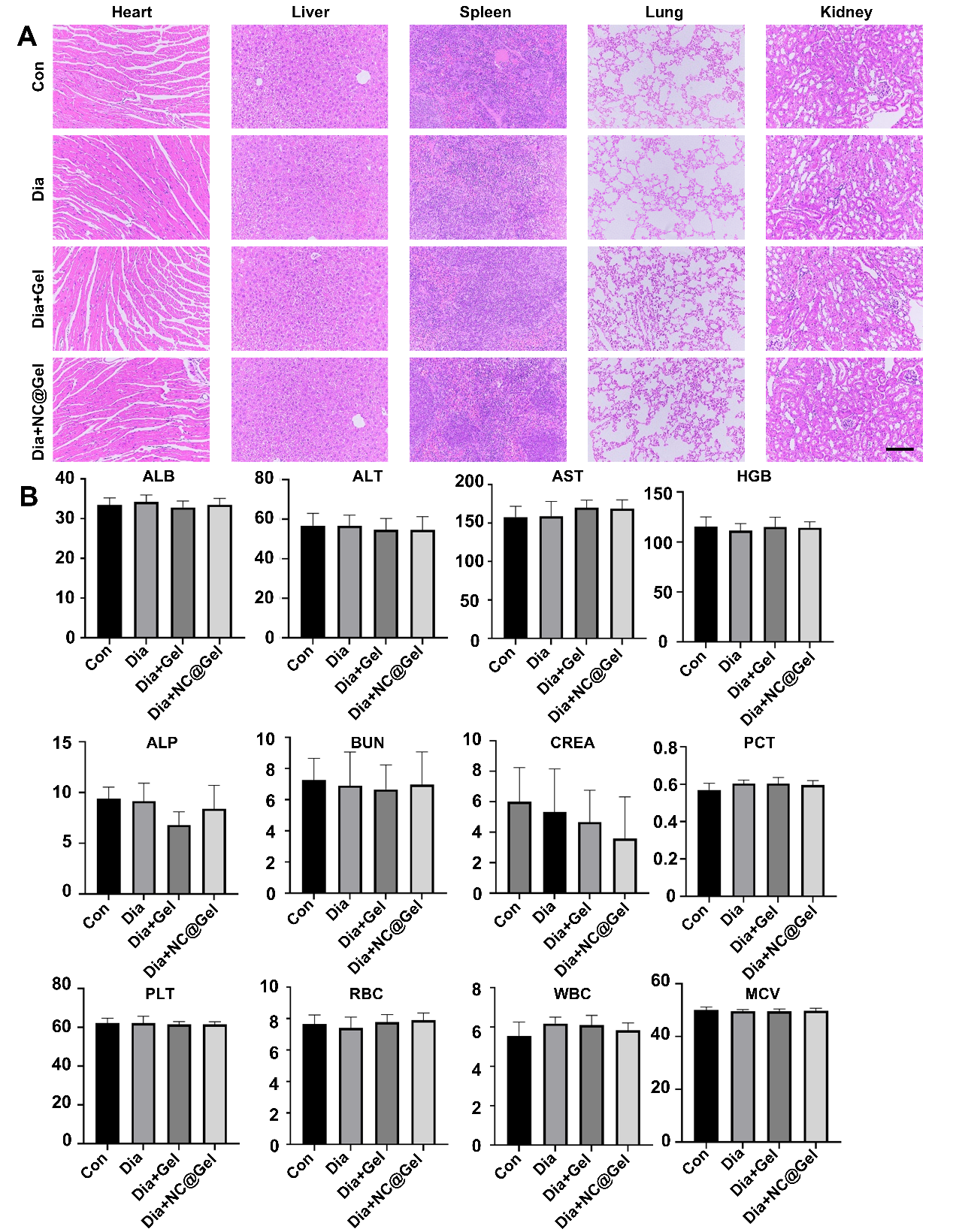
**

Figure S10 *In vivo* biosafety analysis of NC@Gels. (A) Hematoxylin and eosin staining of major organs. Scale bar: 50 μm. (B) Routine blood and biochemical analysis conducted using blood samples collected 1 week after treatment (ALB, Albumin; ALT, Alanine aminotransferase; AST, Aspartate aminotransferase; HGB, Hemoglobin; ALP, alkaline phosphatase; BUN, Blood urea nitrogen; CREA, creatinine; PCT, Plateletcrit; PLT, Platelet count; RBC, Red Blood Cell; WBC, white blood cell; MCV, Mean corpuscular volume. n=3).
